# Qualitative assessment of the primary care outcomes questionnaire: a cognitive interview study

**DOI:** 10.1186/s12913-018-2867-6

**Published:** 2018-02-01

**Authors:** Mairead Murphy, Sandra Hollinghurst, Chris Salisbury

**Affiliations:** University of Bristol, Centre for Academic Primary Care, School of Social and Community Medicine, Canynge Hall, 39 Whatley Road, Bristol, BS8 2PS UK

**Keywords:** Questionnaires, Primary care, Patient-reported outcomes, Patient satisfaction, Patient-Centred care, Family practice, Cognitive interviews, Verbal probing, Think-aloud, Face validity

## Abstract

**Background:**

The Primary Care Outcomes Questionnaire (PCOQ) is a new patient-reported outcome measure designed specifically for primary care. This paper describes the developmental process of improving the item quality and testing the face validity of the PCOQ through cognitive interviews with primary care patients.

**Methods:**

Two formats of the PCOQ were developed and assessed: the PCOQ-Status (which has an adjectival scale) and the PCOQ-Change (which has the same items as the PCOQ-Status, but a transitional scale). Three rounds of cognitive interviews were held with twenty patients from four health centres in Bristol. Patients seeking healthcare were recruited directly by their GP or practice nurse, and others not currently seeking healthcare were recruited from patient participation groups. An adjusted form of Tourangeau’s model of cognitive processing was used to identify problems. This contained four categories: general comprehension, temporal comprehension, decision process, and response process. The resultant pattern of problems was used to assess whether the items and scales were working as intended, and to make improvements to the questionnaires.

**Results:**

The problems identified in the PCOQ-Status reduced from 41 in round one to seven in round three. It was noted that the PCOQ-Status seemed to be capturing a subjective view of health which might not vary with age or long-term conditions. However, as it is designed to be evaluative (measuring change over time) as opposed to discriminative (measuring change between different groups of people), this does not present a problem for validity. The PCOQ-Status was both understood by patients and was face valid. The PCOQ-Change had less face validity, and was misunderstood by three out of six patients in round 1. It was not taken forward after this round.

**Conclusions:**

The cognitive interviews successfully contributed to the development of the PCOQ. Through this study, the PCOQ-Status was found to be well understood by patients, and it was possible to improve comprehension through each round of interviews. The PCOQ-Change was poorly understood and, given that this corroborates existing research, this may call into question the use of transitional questionnaires generally.

**Electronic supplementary material:**

The online version of this article (10.1186/s12913-018-2867-6) contains supplementary material, which is available to authorized users.

## Background

Primary care has evolved in recent years to meet changing population and service needs as well as public expectations. As primary care services globally contend with aging populations and increasing multimorbidity [1], there have been sustained local and national endeavours to improve service quality, costs, and outcomes in primary care. Recent innovations include electronic consultations [2], health coaching and behavioural change therapies [3], and interventions that address needs of frequent attenders [4].

Assessing the effectiveness of primary care interventions from a patient perspective involves the use of patient-reported outcome measures (PROMs). An ‘outcome’ reflects a change in patient health status, knowledge or behaviour, which is attributable to preceding healthcare [5], and PROMs provide important evidence about this change as experienced by the patient [6]. Primary care requires a *generic* PROM, which can be administered across a population, regardless of presenting problem. Many generic PROMs are limited to consideration of symptoms and function, but primary care patients frequently present with problems not causing symptoms or affecting function [7], and many have long-term chronic conditions. Thus leading generic PROMs such as the SF-36 [8] and EQ-5D [9] often show no change following interventions in primary care [10–12]. Other PROMs, designed specifically to measure outcomes in primary care, also have shortcomings. The Measure Yourself Medical Outcome Profile (MYMOP) is an individualised PROM [13] which allows patients themselves to specify their problems and thus shows change when other PROMs do not [12]. However, this measure is administered through interviews, which makes it unfeasible in many trials. It also remains limited to symptoms and function. In contrast, the Patient Enablement Measure (PEI) encompasses broader outcomes that relate to coping, understanding and confidence in health [14] but although it has been validated for primary care [15], it has a transitional format [16] designed to measure outcomes following a single consultation with a physician. For many patients, outcomes will become apparent only after a longer episode of care [17]. Such outcomes may be multi-layered, capturing aspects of enablement, resilience, symptoms and function, and health perceptions.

**Fig. 1:**
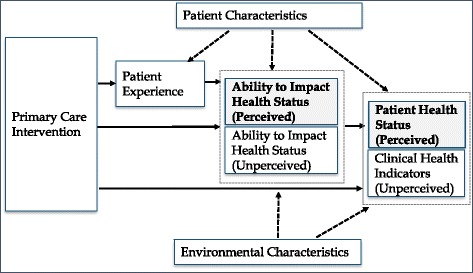
Conceptual model of outcomes influenced by primary care

The Primary Care Outcomes Questionnaire (PCOQ) was designed to fill the gap in evaluative instruments for primary care, by measuring outcomes patients want from primary care and which clinicians can influence [18]. It was developed according to best practice standards [19–21] in a five phase process: a qualitative study to establish the construct [22]; a structured literature review to catalogue existing PROMs which measure this construct; a Delphi consensus process to agree the content; [23] item and scale development through cognitive interviews; and finally a quantitative study [24]. The whole process was underpinned by a conceptual model of outcomes which included patient health status and ability to impact health status (see Fig. 1). The qualitative study identified four inter-related types of outcome: health status outcomes such as symptoms, medication side-effects and the impact of symptoms on patients’ lives; internal health empowerment outcomes such as understanding and ability to self-care; external empowerment such as confidence in seeking healthcare, and access to support; and patient’s perceptions of their health, such as health concerns, and confidence that they are on the right path to dealing with their health conditions. Taken together, these four domains have much in common with the concept of health capability, defined as combining health agency (an individual’s ability to achieve health goals and act as agents of their own health) and health functioning (the outcome of actions to maintain or improve health) [25]. However, they focus on those aspects of health capability which are capable of being influenced by primary care.

The pilot version of the PCOQ was developed in consultation with an advisory group, who checked items for content validity against the constructs identified in the qualitative study [22].

In this paper, we report on the fourth phase in the development process of the PCOQ: improving the item quality and testing face validity through cognitive interviews. Prior to conducting the study, a glossary of items was written, containing a definition of the intended meaning of each item (see Additional file 1).

### Status and transitional PROMs

Most PROMs capture *status*, at a point in time, as opposed to the outcome of an intervention directly [26], with the difference between two status values captured at baseline and post-intervention used to calculate the outcome. A small number of PROMs capture outcome directly without the need for a baseline, using a “transitional” scale. These rely on the patient remembering their health status before the intervention, and assessing their level of change. For example, a common generic transitional item is “thinking about the main problem you consulted your doctor with, is this problem…”, with response options given on a five-point Likert scale from very much better – very much worse [27]. Through our prior structured review, we had identified three transitional instruments which showed higher levels of responsiveness than other PROMs in primary care [14, 28, 29]. We therefore developed both a status and a transitional PROM, and called these the PCOQ-Status and the PCOQ-Change.

Item quality can be improved by assessing whether patients understand the items and whether their responses are appropriate. Face validity is the extent to which a questionnaire appears to be measuring what it is intended to measure: i.e. if it can be taken at face-value [21]. The purpose of this study was to improve the item quality and test the face validity of the PCOQ-Status and PCOQ-Change through cognitive interviews.

## Methods

### Participant recruitment

Patients were recruited from four health centres with a range of deprivation scores in Bristol. Two methods were used to identify patients: those seeking healthcare were recruited by GPs and practice nurses, and those not currently seeking healthcare were recruited by patient participation groups (PPGs) attached to the practices. Patients were provided with an information leaflet, a pre-paid envelope and a return slip containing contact details, age, education, ethnicity and date since last GP appointment. Sampling was purposive (as opposed to random). We purposively sampled to ensure that patients aged over 75 years, ethnic minorities, and people without higher education were all represented. Research shows these groups may have unexpected interpretations or find it more difficult to complete questionnaires [30, 31].

### Data collection

The interviews were conducted in three rounds, with the questionnaire adjusted at the end of each round in response to problems identified. A cognitive interview round was considered completed when there were clear problems identified with a number of questions. We aimed for six to eight interviews per round. Participants were interviewed only once, so each round was carried out on different individuals.

**Fig. 2:**
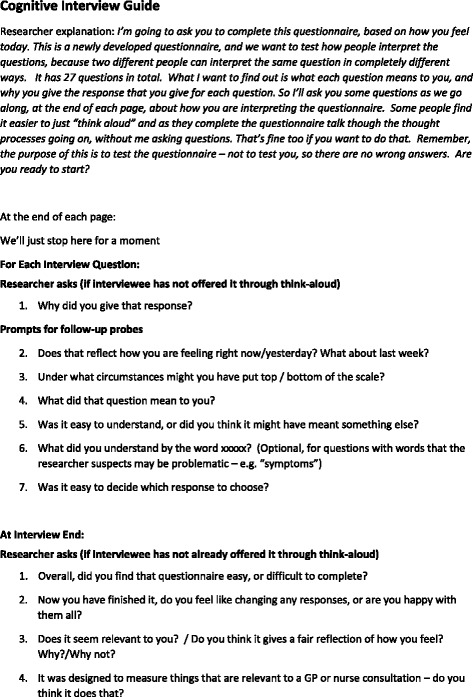
Topic Guide for the Cognitive Interviews

The interviews were conducted by the first researcher (MM), who had received training in qualitative research and cognitive interviews, and had previous experience of cognitive interviewing. Interviews were conducted using immediate retrospective probing [32]. This involved participants completing the questionnaire one page at a time, with a cognitive interview conducted at the end of each page. The main purpose of the cognitive interviews was to improve the item quality of the questionnaires, by uncovering the cognitive processes patients used to answer the question items. The researcher used a single scripted [33, 34] probe “why did you give that response?” for every item. Further probes, both scripted and spontaneous [33, 34] were used as necessary. Face validity was assessed by directly asking patients if they thought their responses provided a true reflection of their current health status, and whether it contained items which were relevant to a primary care consultation. The topic guide is shown in Fig. 2. The interviews were audio-recorded.

**Fig. 3:**
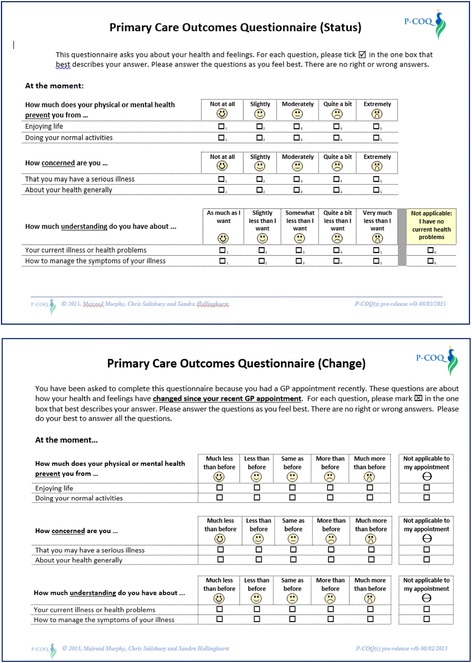
PCOQ-Status and PCOQ-Change, example items from pilot version

The PCOQ-Status is scored on a 5-point unipolar adjectival scale (no problems to extreme problems). The scale wording varies according to the attribute, as determined by the qualitative study [22]. The recall period used of “at the moment” was adopted from the ICECAP [35] and was intended to be interpreted as “that day”, or “within the onset of the current problem”. Cognitive interviews have shown this is more acceptable than the recall period of “today” as used, for example, in the EQ-5D, which some patients find too specific [36] and some ignore altogether [37]. The PCOQ-Change is scored on a 5-point transitional scale, from “much better” to “much worse” with a neutral midpoint. The question items are identical to the PCOQ-Status and the recall period is change from the last GP appointment to the present. Some example items from the pilot version of both questionnaires are shown in Fig. 3.

### Data analysis

Data were coded and analysed using Tourangeau’s model [38], adjusted in response to early interview findings. Tourangeau’s theory, which was further developed by Willis [33], identifies four cognitive tasks required when responding to a questionnaire: comprehension, retrieval, decision and response. The retrieval process, which refers to how information is retrieved from memory, was not relevant for the PCOQ-Status, as it refers to the current time. We replaced retrieval with a process we called “temporal comprehension” as follows.

1. **General Comprehension:** Does the respondent understand the question?
2. **Temporal Comprehension:** Does the respondent understand that the question is referring to the current period?
3. **Decision process:** How does the respondent decide on the answer, for example, do they have a hidden agenda, do they give sufficient mental effort to the task, or do they want to give a socially desirable answer?
4. **Response process**: Does the respondent manage to map their desired response onto the scale without introduction of error? For example, do they understand the scale, and are the scale responses available appropriate?

Verbal reports were summarised in a tabular format by the first researcher (MM). If a problem was identified, the researcher mapped this to one or more of the cognitive processes using memos and verbatim quotes to justify the decision. After each round, these tabulated problems, memos and quotes were reviewed jointly by the three authors (MM/SH/CS), in the context of the glossary of items (see Additional file 1) and adjustments to the questionnaire were agreed based on these identified problems.

As well as the identification of problems in relation to each of the processes in Tourangeau’s model, the data were analysed to identify general issues relevant to patient interpretation of the PCOQ. This was done by close reading of the qualitative interviews and searching for common themes within them.

A second researcher (the “independent coder”), then independently coded four interviews. This was done based on the audio recordings and the glossary of items, without sight of the first researcher’s coding. Both sets of codes were compared in STATA10 and an overall percentage agreement and Cohen’s kappa coefficient calculated. The kappa coefficient (κ) is a measurement of the agreement between two raters for a series of items with dichotomous ratings. If the raters’ agreement is no greater than what would be expected by chance then κ = 0. Kappa scores of 0.75 or higher are generally considered to be excellent, 0.6–0.75 substantial/good and 0.4–0.6 moderate/fair [16].

More details on the study method can be found in a completed COREQ checklist [39] which is attached as Additional file 2.

## Results

### PCOQ-status

#### Summary of results

The identification of problems and adjustment of the PCOQ-Status is presented in Tables 1 and 2. Table 1 shows the problems identified in each round, by participant and cognitive process. Most problems were identified with the comprehension process and the response process. Table 2 (which uses a format adapted from Watt et al. [32]) shows how the problems were reduced in each round by adjusting the items. The table shows the original wording, and the final wording of each item. The columns in between show the number of problems identified in each round, and the position of the vertical line shows the point at which a revision took place. Opening clauses are shaded. As Table 2 illustrates, 20 of the original 28 items were adjusted.

**Table 1:** Number of question items with problems for each participant by cognitive process. (Figures in the first four columns show the total number of items which were problematic for that patient in that process. The total problems column sums across the four processes)

	*Cognitive Process*					*Patient Characteristics*					
*Participant*	*Comprehension* ^*1*^	*Temporal Comprehension* ^*2*^	*Decision* ^*3*^	*Response* ^*4*^	*Total Problems*	*Gender*	*Age*	*Ethnic Group*	*Highest Education Level*	*Recruitment Method*	*Long-Term Conditions*
*1*	*4*	*1*	*–*	*4*	*9*	*Female*	*75+*	*White British*	*O level*	*PPG* ^*a*^	*None*
*2*	*1*	*–*	*–*	*–*	*1*	*Female*	*55–64*	*White British*	*Other* ^*b*^	*PPG*	*> one*
*3*	*4*	*–*	*–*	*3*	*7*	*Female*	*75+*	*White British*	*None*	*PPG*	*None*
*4*	*4*	*–*	*1*	*–*	*5*	*Male*	*35–54*	*White British*	*A level*	*GP*	*> one*
*5*	*2*	*–*	*–*	*1*	*3*	*Female*	*75+*	*White British*	*Other*	*PPG*	*One*
*6*	*6*	*–*	*–*	*–*	*6*	*Male*	*75+*	*Other*	*Other*	*GP*	*> one*
*7*	*1*	*1*	*2*	*6*	*10*	*Male*	*18–34*	*Other*	*NVQ*	*GP*	*One*
*Round 1 Total*	*22*	*2*	*3*	*14*	*41*						
*8*	*2*	*–*	*1*	*4*	*7*	*Female*	*35–54*	*White British*	*None*	*GP*	*one*
*9*	*2*	*2*	*–*	*–*	*4*	*Female*	*18–34*	*White British*	*NVQ*	*GP*	*> one*
*10*	*2*	*3*	*–*	*–*	*5*	*Male*	*35–54*	*Other*	*Degree*	*GP*	*none*
*11*	*–*	*–*	*–*	*1*	*1*	*Male*	*55–64*	*White British*	*Not given*	*GP*	*one*
*12*	*–*	*–*	*–*	*3*	*3*	*Male*	*55–64*	*White British*	*A level*	*PPG*	*one*
*13*	*3*	*–*	*–*	*–*	*3*	*Female*	*35–54*	*White British*	*None*	*GP*	*one*
*14*	*0*	*–*	*–*	*–*	*–*	*Female*	*65–74*	*White British*	*O level*	*GP*	*> one*
*Round 2 Total*	*9*	*5*	*1*	*8*	*23*						
*15*	*–*	*–*	*–*	*1*	*1*	*Female*	*35–54*	*White British*	*NVQ*	*GP*	*> one*
*16*	*2*	*–*	*–*	*–*	*2*	*Male*	*65–74*	*White British*	*Other*	*GP*	*> one*
*17*	*–*	*–*	*–*	*–*	*–*	*Female*	*35–54*	*White British*	*O Level*	*GP*	*one*
*18*	*1*	*–*	*–*	*1*	*2*	*Male*	*65–74*	*White British*	*Not given*	*PPG*	*none*
*19*	*2*	*–*	*–*	*–*	*2*	*Female*	*55–64*	*White British*	*Other*	*PPG*	*> one*
*20*	*–*	*–*	*–*	*–*	*–*	*Male*	*18–34*	*White British*	*NVQ*	*PPG*	*none*
*Round 3 Total*	*5*	*–*	*–*	*2*	*7*						

^1^Comprehension: (has the question been understood?)

^2^Temporal Comprehension: (has the participant correctly understood the period as current?)

^3^Decision Process (was any error introduced when the participant decided what response to give?)

^4^Response Process (Did the response categories include the desired response?)

^a^Patient Participation Group

^b^Other qualifications included Secretarial, City and Guilds technician, registered nurse and nursery nurse

**Table 2:**
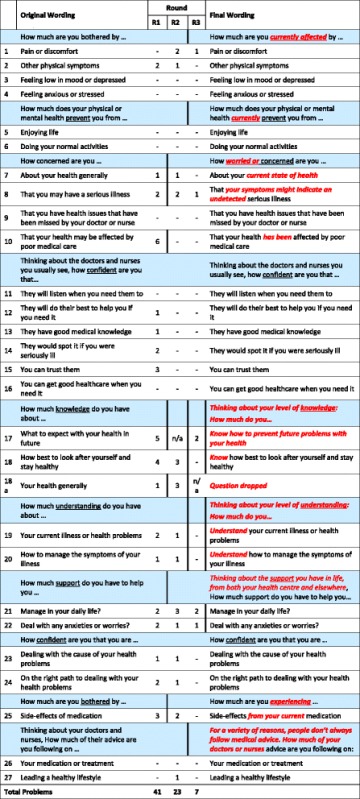
Problems by item, and revision of items in each round. (Numbers in each round are the number of problems identified)

Questions 11–16: The wording of these questions was not changed. However, the ordering was changed between round one and round two, and this may have helped comprehension

Questions 17–20: The double lines at the end of round three indicate changes to the wording of the response options, not the item. The first option was changed from “As much as I want” to “I know as much as I want” / “I understand as much as I want”

Questions 21–22: The double lines at the end of round three indicate changes to the wording of the response options, not the item. A “not applicable” option was added at the end of this round. This was discussed with round three participants

#### Comprehension

There were four types of comprehension problem: ambiguous language, failure to comprehend a word, conceptually difficult items, and comprehension resulting from split sentences. The split sentences are part of the PCOQ format, which consists of a list of phrases, qualified by a clause at the beginning. In some questions, participants appeared to forget the qualifying clause by the time they reached the phrase. Through wording and formatting changes, the number of problems was reduced from 22 to five throughout the three rounds. Comprehension problems were not always corrected. For example, P1, gave an incorrect definition of the word “symptoms” on probing. Yet, prior to being directly asked to provide a definition, her explanations suggested she understood the word sufficiently in context to give an accurate response, so this word was not adjusted.

#### Temporal comprehension

Some patients based their response on past rather than current status. For example, P9 responded “quite a bit” to how much she was affected by other physical symptoms, although her most recent symptoms were a bad asthma attack five years ago. She explained, after hesitation,

“If they could have turned round and said ‘how does it affect you now’, I would have turned around and said ‘not at all’ but because it said ‘at the moment’ I was like ‘hang on a minute, which one should I tick?’” P9.

Consideration was given to adjusting the phrasing “at the moment.” However, as described in the methods section, this phrase had been specifically selected as having greater face validity than “today” for many patients so rather than making this change, the words “currently affected” were added in to emphasise the period.

#### Decision process

There were very few problems coded to decision process. The problems identified split into two types. The first was a halo / reverse halo effect [16]. For example, P7, who had a low opinion of his health centre, and who had given relatively negative responses throughout, gave similarly negative responses to the question “how much support do you have to help you manage in your daily life?” On verbal probing, although he understood the intended meaning of the question, he was not able to explain what kind of support he was missing. It seemed to the researcher that he was using the questionnaire as a statement of his opinion of the health centre, rather than this item being a true reflection of his levels of support in life. The second was a social desirability bias. This was in relation to the question “how much of your doctor’s or nurse’s advice are you following in living a healthy lifestyle?” P8, for example, had said earlier in the interview that she often disregarded clinicians’ advice, yet she ticked the option “most of the advice”. This question was changed to include the beginning sentence: “For a variety of reasons, people don’t always follow medical advice. How much of your doctor’s or nurse’s advice are you following on…?” Similar bias may have occurred with other patients but, by its nature, social desirability bias is difficult to detect as it depends on participants making the information available.

#### Response process

Participants generally found it easy to map their decision to the response options. The emoticons and consistent order of response categories (positive to negative) seemed to help with this. Three types of response process issue were raised. Firstly, the “not applicable” option went unnoticed by some participants, and secondly, “not applicable” was not available for some items where participants felt it was needed. Both issues were improved by rewording and reformatting. The third type of problem was a perceived insufficient number of response options: some participants suggested there should be another point on the scale. Because this did not lead to any missing data, and because increasing the number of options would have reduced legibility, the questionnaire was not adjusted.

#### Face validity

Seventeen of twenty participants indicated, in response to a direct question, that the PCOQ-Status reflected their current status. Of the three who thought it did not provide a reflection of their current state, two of these were in round one, and their issues were addressed in later rounds. In general, participants appeared to clearly comprehend the questionnaire, and answered it quickly, taking a median time of 4 min.

#### Other findings

##### Attribution

Some participants hesitated over what aspects of their life to include in their decision-making. For example, P2, who had multiple long-term conditions, had also had a recent fall. At the time she completed the questionnaire, the physical pain, loss of function and concern caused by the fall had a greater influence on her overall health status than her long-term conditions. At times, she paused to ensure she was incorporating both her chronic and acute illness. Nonetheless, she managed to answer the questions giving an holistic view of her health.

##### Adaptation to illness

In some patients, particularly the elderly and those with long-term conditions, the Health Status items seemed to be influenced by expectations and adaptation to illness. For example, P5, an 80-year-old woman who appeared out of breath when walking, scored “not at all” to all questions in the Health Status domain. On probing she said:

P5: I do get breathless if I walk too fast, that’s the only thing.

Int: Why did you put “not at all” in that case?

P5: Because, for my age, I’m very well really. Perhaps I should have done slightly. But I think for my age I’m pretty good.

This reflects that the questionnaire is capturing a subjective view of health status, which is influenced by the patient’s comparison to her peer group and adaptation to illness.

#### Independent coding comparison

The Kappa score for the four independently coded interviews showed excellent overall agreement, with an overall kappa of 75%. Three of the four process-level kappas were good or excellent: comprehension (K = 0.73), temporal comprehension (K = 0.65), response (K = 1.00). For the decision process, three problems were identified by one coder, and none by the other. The kappa score was zero, which will be discussed later.

### PCOQ change

#### Cognitive interview results

Six of the seven participants from round one had recently attended the doctor, and were therefore able to complete the PCOQ-Change. All six participants in round 1 found the PCOQ-Change difficult to complete, and some disengaged from the cognitive interview, as they struggled to explain their reasons for response. This meant that it was not possible to extricate the four aspects of Tourangeau’s cognitive model [38] from the interview data. Comprehension problems were identifiable, but the retrieval, decision and response processes were not. These were replaced by the single category of “struggle”, which has been used in other frameworks [40] to identify patients who hesitated or found difficulty with formulating a response. Table 3 shows instances of comprehension and struggle by participant, and by question. Because the format of the questionnaire was unsuccessful, the PCOQ-Change was not taken forward to a second round.

**Table 3:** Comprehension and struggle problems in the PCOQ-Change

#	Item	C^1^	S^2^
1	Pain or discomfort	1	0
2	Other physical symptoms	1	1
3	Feeling low in mood or depressed	2	0
4	Feeling anxious or stressed	1	0
5	Enjoying life	2	0
6	Doing your normal activities	2	0
7	Worries about health generally	0	0
8	Worries about serious illness	1	1
9	Worries about missed health issues	1	0
10	Worries about damage to health	1	0
11	Clinicians will listen	2	1
12	Clinicians will do their best to help you	2	1
13	Clinicians have good medical knowledge	1	1
14	Clinicians would spot it if you were seriously ill	2	1
15	You can trust clinicians	2	1
16	Can get good healthcare when you need it	2	1
17	Know to expect with your health in future	2	2
18	Know how to look after yourself and stay healthy	2	2
18a	Understand your health generally	1	3
19	Understand current illness	1	0
20	Understand how to manage symptoms of illness	2	1
21	Support to manage in daily life	2	0
22	Support to deal with anxiety or worries	2	0
23	Dealing with the cause of health problems	1	0
24	On the right path to dealing with health problems	1	1
25	Side-effects	3	0
26	Follow clinician advice on medication	2	0
27	Follow clinician advice on lifestyle	2	0
Total		44	17
Participant		C^1^	S^2^
P1		16	
P2		–	11
P4		–	4
P5		–	–
P6		25	
P7		3	2
Total		44	17

^1^C: Comprehension (has the question been correctly understood)

^2^S: Struggle (did the participant hesitate, leave the question blank, or express confusion over what response to give)

#### Comprehension

The major problem with the PCOQ-Change was participants misinterpreting it as being a status questionnaire. Three of the six participants responded at least partly based on their current status, rather than their change in status. This resulted in artificially high scores for P1 and P6, and low scores for P7. For example, P6, whose appointment had been unrelated to pain, hesitated between “much less than before” and “less than before” on the first question on pain. The emoticons added to his confusion as they imply a state, rather than a change and he decided on “less than before” (which had a closed mouth smile) saying “well I’m not laughing, so it’s got to be that one.”

#### Struggle

All participants apart from one hesitated or struggled with some aspects of the PCOQ-Change. Two participants (P1 and P6) were so confused by the questionnaire, that they completed it quickly, but with little apparent thought or understanding after the first page, a process known as “satisficing” [16]. It was, therefore, not possible to accurately document their levels of struggle.

#### Face validity

Four of the six participants thought the PCOQ-Change lacked face validity; that is, they were not clear what it was trying to measure or why. The other two verbally reminded themselves while completing the questionnaire of the period “since the last appointment”.

#### Independent coding comparison

The independent coding showed a low overall kappa score (0.33). The kappa for struggle was good, at 0.65, but the kappa for comprehension was low (0.20). This results partly from the low number of problems identified in the two selected interviews, but also from the extent of misunderstanding and the tendency of participants to disengage from the interview.

## Discussion

### Key findings

The cognitive interviewing was successful in the aim of improving item quality in the PCOQ-Status. The interviews demonstrated that the PCOQ-Status had good face validity and that the PCOQ-Change lacked face validity.

#### PCOQ-status

Participants completed the PCOQ-Status quickly and found it comprehensible and face valid. Cognitive interviewing improved item quality, by reducing the number of problems identified in each round, particularly with comprehension problems.

Our results indicated that the Health Status items, on symptoms and the impact of symptoms on life, were influenced by patient expectations and adaptation to illness. This issue has already been noted in the literature with regard to generic measures of health status. In cognitive testing of the SF-36, Mallinson noted that patients often rated themselves in comparison to their peers, despite the fact that they had been specifically instructed not to do this [41]. Mallinson suggested that there was little consistency of approach among people in this regard, and that the meanings of aggregated SF-36 data were therefore uncertain [41]. However, Mallinson conflated evaluative instruments (measuring change over time) and discriminative instruments (measuring cross-sectional differences between various groups of people) [42]. The SF-36 has been used for both purposes [43], but the primary purpose of the PCOQ is evaluative, therefore consistency between people is less important than consistency in each person between administrations of the instrument.

Initial cognitive interview rounds noted that the Health Perceptions items on concerns, and confidence in the health plan, seemed to be measuring traits rather than states. This has proved the case with other health perceptions questionnaires, which have shown high stability over time [44]. By adjusting the items in an attempt to capture current state, rather than underlying trait, this domain should prove more sensitive to change.

#### PCOQ-change

The issue of misinterpretation of the PCOQ-Change was, to some extent, anticipated. A key problem with transitional scales is that often questionnaire respondents do not accurately recall their baseline health state [16] and they compensate for this by constructing or guessing a response based on their current health state [45]. If they are feeling well, they rate themselves as improved; if feeling unwell, they rate themselves as having deteriorated [27, 46]. Despite this, we piloted the PCOQ-Change because transitional questionnaires are, nevertheless, widely used [47, 48]. Proponents suggest such measures are simply quantifying what clinicians routinely do anyway, and therefore have implicit validity [27]. They also offer the potential for increased responsiveness, exemplified by the PEI [15] and ORIDL [28]. However, such apparent responsiveness may not be a reflection of true change. Unlike change questionnaires, transitional questionnaires do not suffer from ceiling effects at baseline, and therefore always have the capacity to demonstrate change. Yet they often correlate with current status better than they do change measured from baseline, suggesting they may measure a construct which is closer to status than change [16].

### Strengths and limitations of the methodology

This study was carried out with a relatively small number of participants, and was qualitative in nature. The results from qualitative research are always influenced by the social and cultural lens of the researcher [49]. The main researcher was a white, university-educated, British woman with a non-clinical background. To add rigour to the analysis process, she kept detailed memos to reflect on how she was categorising the data, and these were discussed with the co-researchers at the end of each round. The independent coding also increased the rigour of the analysis process.

Tourangeau’s model [38], modified to replace the retrieval category with temporal comprehension, was an effective method for mapping and resolving problems in the PCOQ-Status. The sample interviewed contained patients from a wide-range of ages, educational backgrounds and health status.

Cognitive interviewing is underpinned by the assumption that respondents are able to provide verbal reports of their thought processes. However, the quality of these verbal reports is not often tested [50]. While the method was successful, there were some learning points on the veracity of verbal reports in cognitive interviews.

Previous studies have found cognitive interviews to be most sensitive to comprehension problems [50]. This study, similarly, found substantially more comprehension problems, and also found that these could be reduced through rewording. Previous research has highlighted the potential danger of using scripted probing methods to uncover comprehension problems. Conrad and Blair point out that, for simple questions, the cognitive processes may be so automatic that the verbal reports broken down by the four cognitive processes might not be accessible to interviewees. If interviewees are prompted for explanations when none is available, they are likely to construct a vague response, rather than no response at all. For example, when P1 was probed on the meaning of the word “symptoms” she gave an incorrect definition despite providing a response which reflected the health status she verbally described. In this case, the problem might have been introduced by the probing: she may have understood the term sufficiently in context, but not well enough to provide formal definitions [50].

Unlike the comprehension problems, the response problems did not substantially reduce between rounds. This is because most of them were related to the number of options on the scale. Increasing the number of options on the adjectival scale would have reduced legibility, and research shows that optimal psychometric properties are offered by a four to seven-point scale [21, 51].

As with other studies [40, 52], very few problems were found with the decision process. The kappa score was zero but one coder did not identify any problems. The kappa statistic generates artificially low scores when very low numbers of items are observed. The low agreement probably also arose because decision processes are, by nature, hidden and depend more than the other areas on the judgement of the researcher [50]. Willis suggests that this should include an assessment of whether the respondent has given “sufficient mental effort” [33] (pg. 2). This, however, is a highly subjective decision. It also includes whether the person has tried to give a socially desirable response. But unless this is exposed in the interview (such as the woman who mentioned her poor adherence early in the interview, but then gave a positive response to the adherence question), social desirability is difficult to uncover. This kind of hidden decision process is sometimes described as “faking good” [31]. The opposite decision process “faking bad” [31] may also have been present, but hidden. For example, although it was coded as a comprehension problem when P8 indicated bothersome symptoms based on her past, not current, health state, it is possible that it was a hidden decision process. For the purposes of adjusting the questionnaire, the categorisation is of more than academic interest. Provided the problem was one of temporal comprehension, the correction which was made to the wording should rectify the problem: (from “how much are you bothered by pain or discomfort” to “how much are you currently affected by pain or discomfort”). However, if the issue was one of decision process: that the participant understood the question, but based her decision about which response to give on her wish to convey herself as a sick person, no amount of wording change would rectify this.

## Conclusions

Overall the method of cognitive interviewing proved successful in improving the item quality of the PCOQ-Status. Some of the findings have general implications for qualitative testing of questionnaires. Cognitive interviews often find low numbers of problems with the decision process. [40, 52] However this is not always attributed to the fact that these processes are hidden, and therefore may manifest as comprehension problems instead. Temporal comprehension is not normally identified as a separate process, but given that in both this study and other studies [37], patients often use an incorrect time reference, the isolation of these as distinct problems in future could greatly improve the face validity of questionnaires.

This research found that the PCOQ-Change was poorly understood by patients. Given that this corroborates existing research [16], this may call into question the use of transitional questionnaires for measurement of outcome in primary care. Certainly, it points to a need for greater cognitive testing of transitional questionnaires, as many of these have been quantitatively tested [15, 28], but had limited or no testing through cognitive interviews. Reporting the results of psychometric testing without first carrying out cognitive interviews may mask the systematic bias created by some patients answering transitional questions based on their current status, rather than their change in status.

The PCOQ-Status was well understood by patients, and the number of problems reduced through each round. It was found to capture a subjective view of health, suggesting it would be suitable as an evaluative, as opposed to discriminative instrument [42]. Unlike instruments which have not been cognitively tested, the results of future quantitative psychometric testing can now be confidently interpreted in the context of clear and comprehensible items with demonstrated face validity. These have been established using rigorous methods, and the instrument subject to detailed scrutiny through these cognitive interviews.

## Additional files

Additional file 1: Word Document Primary Care Outcomes Questionnaire Construct: Glossary of Items. (DOCX 18 kb)

Additional file 2: Word Document Consolidated criteria for reporting qualitative studies (COREQ): 32-item checklist. (DOCX 23 kb)

## Abbreviations

ASCOT: Adult Social Care Outcome Tool
COREQ: Consolidated criteria for reporting qualitative research
EQ-5D: EuroQol-5 dimensions
GP: General Practice / General Practitioner
ICECAP: ICEpop CAPability measure
ORIDL: Outcomes Related to Impact on Daily Living
PCOQ: Primary Care Outcomes Questionnaire
PEI: Patient Enablement Instrument
PPG: Patient Participation Group
PREM: Patient Reported Experience Measure
PROM: Patient Reported Outcome Measure
SF-36: Medical Outcome Study Short Form 36
