# Supplementary material for: Qualitative assessment of the primary care outcomes questionnaire: a cognitive interview study
Source: BMC Health Serv Res. 2018 Feb 1;18:79. doi: 10.1186/s12913-018-2867-6 (PMC5796473; doi:10.1186/s12913-018-2867-6)
Supplement: Supplementary file 1 — Word Document Primary Care Outcomes Questionnaire Construct: Glossary of Items. (DOCX 18 kb) [file 12913_2018_2867_MOESM1_ESM.docx]

**Primary Care Outcomes Questionnaire Construct: Glossary of Items**

This table gives an item-level description of the primary care outcomes questionnaire. The purpose of this is to document the intended meaning of the items, so that these can be compared to patient interpretations in cognitive interviews, and problems can be consistently coded between multiple coders.

| **#** | **Wording** | **Explanation** |
| --- | --- | --- |
|  | At the moment | All questions are trying to assess the person’s current state. For most people, this will be over the past day or two.  Explanatory notes: We discussed use of “today”, “over the last two days”, “at present” and “at the moment” to capture current state during questionnaire development. We decided on the phrase “at the moment”, because the exact period might vary for some patients, depending on their condition. To use the example of pain / symptoms: if we used the phrase “today” or “now”, this might result in patients who had pain on walking, or symptoms in the evenings to indicate no problems, when in fact, they did have daily pain / symptoms. While “over the last two days” might work for these patients, it is not a time-period often used in questionnaires, and may present confusion. In summary, we intend the questionnaire to measure current state, and any patients reflecting past state (e.g. indicating problems with pain or symptoms which happened some time ago and are now under control) should be logged as having problems with their understanding of the question. |
|  |  |  |
| 1 | How much are you bothered by … pain or discomfort | This question is intended to capture the person’s subjective assessment of their own current levels of pain or discomfort. |
| 2 | How much are you bothered by … other physical symptoms | This question is intended to capture the person’s subjective assessment of current physical symptoms. This might include cough, itching, breathlessness, fatigue or any other physical symptoms. |
| 3 | How much are you bothered by … feeling low in mood or depressed | This is intended to capture the person’s subjective assessment of their current depression levels. The term “low in mood” was used as well as depression to capture those people who were feeling slightly down, but might not have a diagnosis of depression. |
| 4 | How much are you bothered by … feeling anxious or stressed | This is intended to capture the person’s subjective assessment of their current anxiety or stress levels. Patients should be able to distinguish this from the previous question, even if they give the same response to both. |
| 5 | How much does your physical or mental health prevent you from … enjoying life | This is intended to capture the extent to which physical or mental health problems currently stop patients enjoying life. This could be through symptoms ‘niggling’ them on a daily basis, or from impaired function preventing them from carrying out activities which they enjoy or used to enjoy. |
| 6 | How much does your physical or mental health prevent you from … doing your normal activities | This is intended to capture the extent to which patients are currently able to continue their normal life as far possible without disruption from their illness or symptoms. ‘Normal’ activities is a subjective construct. It may include paid employment, leisure activities, housework/DIY, shopping or anything involving acceptable levels of physical and mental function. |
| 7 | How concerned are you … that you may have a serious illness | This is intended to capture the extent to which patients are currently concerned that their signs or symptoms indicate a serious illness. The emphasis is intended to be undiagnosed serious illness, or unforeseen complications from existing illnesses. |
| 8 | How concerned are you … about your health generally | This is intended to capture the extent to which patients are currently concerned about their health in general. |
| 9 | How concerned are you … that your health may be affected by poor medical care? | This question is intended to capture whether patients believe that they currently have health problems which have come about as a result of poor medical care, delays in care or missed diagnosis, at some point in the past. |
| 10 | How concerned are you … that you have health issues that have been missed by your doctor or nurse. | This question is intended to capture patients’ current levels of concern about health problems which have potentially been missed by healthcare practitioners. It should capture their current beliefs on the possible causes of their signs or symptoms and whether they are convinced that they have been thoroughly investigated. |
|  |  |  |
|  | Thinking about the doctors and nurses you usually see | This phrase is intended to focus the patients mind on the doctors and nurses currently responsible for their care. For most patients, this will be the doctors and nurses at the general practice, although for some patients it might also include secondary care. |
| 11 | How confident are you that … they have good medical knowledge | This question is intended to capture the patient’s beliefs about their doctors’ and nurses’ medical knowledge. |
| 12 | How confident are you that … they would spot it if you were seriously ill | This question is intended to capture the patient’s confidence that the doctors/nurses would correctly and promptly diagnose them, in particular if they have an underlying serious illness. |
| 13 | How confident are you that … you can trust them | This question is intended to capture patient’s trust in their doctors and nurses. Trust is a wide concept, encompassing aspects the clinician’s professionalism, integrity, openness and commitment to confidentiality and patient care. |
| 14 | How confident are you that … they will do their best to help you if you need it | This question is intended to capture patient’s belief that their doctors and nurses are committed to helping them if they need it. This includes the patient perception of the clinician’s compassion, commitment to the patient, and whether the clinician focuses on the patient’s needs and concerns, rather than an exclusive bio-medical agenda. |
| 15 | How confident are you that … they will listen when you need them to. | This question is intended to capture patient’s belief that they will be listened to by their health professionals. This includes having access to a clinician who they believe to be interested in hearing all the patients problems/concerns, and being given sufficient time to cover all their concerns within individual appointments. |
| 16 | How confident are you that … you can get good healthcare when you need it. | This question is intended to capture patient’s belief that they can access good healthcare when they need it. This encompasses aspects of service availability (can the patient get a timely appointment), confidence in clinicians (can they access a clinician they have confidence in) and candidacy (do they perceive the GP gatekeeping role facilitates or prevents them from accessing the services they believe they need and deserve) |
|  |  |  |
|  | Scale from “as much as I want – none of the X that I want” | Questions 17 – 23 incorporate patient desire into the scale. This is because this set of outcomes was characterised by deviant cases in the qualitative work, in that they were important to a large number of patients, but to some patients, they were of no importance. For example, “understanding” of health problems was extremely important to a large number of patients, who felt both empowered and reassured through increased knowledge of their conditions. In contrast, some patients with fairly low levels of understanding did not want to increase their knowledge further, preferring instead to trust in their clinicians. The cognitive interviews should test the extent to which patients understand these scales as intending to reflect whether they are satisfied with their levels of these constructs, as opposed to whether they meet an external benchmark. |
| 17 | How much knowledge do you have about … how best to look after yourself and stay healthy | This question is intended to capture the extent to which patients feel they know enough about remaining healthy and/or improving their health. This is both with regards to a general healthy lifestyle, and with regard to specific conditions or illnesses: whether they feel they have enough information to make the right lifestyle choices for their health. Those patients who do not feel they have a knowledge gap should indicate the top score. |
| 18 | How much knowledge do you have about … what to expect with your health in the future | This question is intended to capture the extent to which patient clearly understand the future implications of any health conditions they have. It should reflect their understanding of the likely prognosis of their illness or illnesses. Those patients who do not feel they have a knowledge gap should indicate the top score. |
| 19 | How much knowledge do you have about … your health generally | This question is intended to capture the extent to which patients understand what is happening currently with their health. It should reflect their understanding of any illness diagnoses, signs or symptoms. Those patients who do not feel they have a knowledge gap should indicate the top score. |
| 20 | How much support do you have to help you … manage in your daily life | This question is intended to capture the extent to which patients feel they have enough support to help them manage in daily life. This could include healthcare support, social support or financial supports. This support could come from healthcare services, social services, family, friends or other services. Those patients who do not feel they need any more support should indicate the top score. |
| 21 | How much support do you have to help you … deal with any anxieties or worries | This question is intended to capture the extent to which patients feel they have enough support to help them deal with anxiety or worry. This support could come from healthcare services, social services, family, friends or other services. Those patients who do not feel they need any more support should indicate the top score. |
| 22 | How much understanding do you have about … your current illness or health problems | This question is intended to capture whether patients have as much understanding as they want about their current health problems. Because the response scale incorporates patient desire, those patients to whom this is not important should indicate the top score, or the not applicable score. |
| 23 | How much understanding do you have about … how to manage the symptoms of illness | This question is intended to capture whether patients have as much understanding as they want about how to mitigate or alleviate the symptoms of illness. Because the response scale incorporates patient desire, those patients to whom this is not important should indicate the top score, or the not applicable score. |
| 24 | How confident are you that you are … dealing with the cause of your health problems | This question is intended to capture the extent to which patients believe that their health issues are being tackled in the right way by their clinicians and themselves. In incorporates their belief that they have a correct diagnoses, and that their clinicians have given due consideration to how to deal with these health problems, rather than offering a ‘quick- fix’. |
| 25 | How confident are you that you are … on the right path to dealing with your health problems | This question is intended to capture the patients confidence that that their health issues are being deal with in the right way, and that, where they need ongoing care or services outside primary care, that this is being facilitated. |
| 26 | How much are you bothered by …side-effects of medication | This question is intended to capture patient’s current level of bother from side-effects of medication. This should be medication that they are currently taking, and side-effects that they have recently experienced. |
| 27 | Thinking about your doctors and nurses, how much of their advice are you following on … your medication or treatment | This question is intended to capture the extent to which patients are adhering to their medication regimen as prescribed by their doctors/ nurses. For most patents, these doctors/nurses will primary care clinicians, but for some it might include secondary care for a few. |
| 28 | How much of their advice are you following on … leading a healthy lifestyle | This question is intended to capture the extent to which patients are following any advice on healthy lifestyles given by their doctors and nurses. This might include general advice on diet and exercise, or specific advice relevant to the patient and their long term condition(s) |
