# Supplementary material for: Qualitative assessment of the primary care outcomes questionnaire: a cognitive interview study
Source: BMC Health Serv Res. 2018 Feb 1;18:79. doi: 10.1186/s12913-018-2867-6 (PMC5796473; doi:10.1186/s12913-018-2867-6)
Supplement: Supplementary file 2 — Word Document Consolidated criteria for reporting qualitative studies (COREQ): 32-item checklist. (DOCX 23 kb) [file 12913_2018_2867_MOESM2_ESM.docx]

| Consolidated criteria for reporting qualitative studies (COREQ): 32-item checklist^1^ | |
| --- | --- |
| 1. Interviewer/facilitator | The cognitive interviews were conducted by Mairead Murphy. |
| 1. Credentials | Mairead Murphy and Sandra Hollinghurst both have PhDs. Chris Salisbury has an MD and is a FRCGP. Beth Simmonds (who is acknowledged, but not an author, and carried out the independent coding) has a PhD in qualitative research. |
| 1. Occupation | All authors are researchers at the University of Bristol. Chris Salisbury is a professor of primary healthcare, Sandra Hollinghurst is a senior lecturer in health economics, Mairead Murphy is a senior research associate. Beth Simmonds is a lecturer at University of Portsmouth. |
| 1. Gender | Chris Salisbury is male and the other researchers are female. |
| 1. Experience and Training | Mairead Murphy has undergone training in research governance, qualitative research and cognitive interviewing at the University of Bristol. Chris Salisbury and Sandra Hollinghurst both have more than 20 years’ experience in health services research. |
| 1. Relationship established | Prior to study commencement, the interviewer and the participants had no previous contacts. Rapport was built before the cognitive interview started through the researcher explaining the study, answering any questions from participants and taking informed consent. |
| 1. Participant knowledge of the interviewer | The participants did not have prior knowledge of the interviewer before the study. When participants were recruited (by either the health centre nurse, GP or PPG group convener) they were provided with an information leaflet about the study, which also provided some information about the researcher (Mairead Murphy) including contact details. Mairead Murphy explained her background, and the reason for doing the study prior to the start of the interview. |
| 1. Interviewer characteristics | The main researcher was a white, university-educated British woman with a non-clinical background. Qualitative research is alway influenced by the perspective of the researcher, but these characteristics should not have added particular bias. To add rigour to the analysis process, she kept detailed memos to reflect on how she was categorising the data, and these were discussed with the co-researchers at the end of each round, in the context of the glossary of items. The independent coding also increased the rigour of the analysis process. |
| 1. Methodological orientation and Theory | The key methodological framework used was Tourangeau’s model of cognitive processing.^2^ ^3^ Within this, a framework analysis approach was taken, so problems were coded within the four categories in this framework (general comprehension, temporal comprehension, response process, decision process) |
| 1. Sample | The participants were identified by GPs and nurses (patients seeking healthcare) and Patient Participation Group (PPG) conveners (patients not seeking healthcare).  Patients were then purposively sampled by the researcher to ensure that patients aged over 75 years, ethnic minorities, and people without higher education were all represented. Research shows these groups may have unexpected interpretations or find it more difficult to complete questionnaires.^4 5^ |
| 1. Method of approach | The participants were approached by GPs and nurses at the end of the consultation, or by the PPG convener at a PPG meeting or via email. Interested patients returned their form to the researcher (Mairead Murphy) who then purposively sampled patients to contact directly for interview. |
| 1. Sample Size | 20 |
| 1. Non-participation | Non-one dropped out, as the study was cross-sectional. We have no details on non-participation (i.e. the patients who were approached by GPs/nurses or PPI conveners, but declined to send their details to the researcher.) |
| 1. Setting of Data Collection | Interviews were conducted in a room at the University of Bristol, or the patient’s health centre, depending on the patient’s preference. |
| 1. Presence of non-participants | No |
| 1. Description of the sample | This is described in table 1 of the manuscript. |
| 1. Interview guide | A brief interview guide was used. This is attached as Figure 2. This was pilot tested with two University of Bristol colleagues. |
| 1. Repeat interviews | We did not carry out repeat interviews (with the same participants) because this was not appropriate for our research design. Cognitive interviews are carried out in rounds, with different participants in each round. Using the same participants in each round would give biased results, as participants second completion would be reactive to (i.e. influenced by) the first cognitive interview. |
| 1. Audio-/visual recording | We used audio recording to collect the data. |
| 1. Field notes | Field notes were made during the interviews to note participant expression, or other non-verbal cues during questionnaire completion. |
| 1. Duration | The interviews lasted an average of 39 minutes in round 1 (when two questionnaires were tested) and 30 minutes in rounds 2 and 3 (when only one questionnaire was tested). |
| 1. Data Saturation | Data saturation was achieved by round 3 as no new problems were being identified at that stage. All problems identified in round 3 had already been identified by patients in rounds 1 and 2. Some were issues that the researcher decided not to change, in recognition of the fact that no questionnaire is perfect, and rectifying problems may introduce different problems: e.g. some respondents wanted to give a response between slightly and moderately, but introduction of more response options would have required double-banking, which is visually unclear and unappealing. |
| 1. Transcripts returned | Transcripts were not returned to participants for comment or correction. |
| 1. Number of data coders | 2 |
| 1. Description of the coding tree | Yes, the four dimensions of the adjusted Tourangeau model^2^ ^3^ i.e. general comprehension, temporal comprehension, response process and decision process) were used as a framework to categorize problems with the questionnaire. |
| 1. Derivation of themes | Themes were derived from the data as well as from the theoretical model of Tourangeau. |
| 1. Software | Excel and Word were used to manage the thematic data |
| 1. Participant checking | No |
| 1. Quotations presented | Yes, participant quotations are presented to illustrate the themes. |
| 1. Data and findings consistent | Yes. Data shows reduction in number of problems over the three rounds, which is consistent with the overall findings. |
| 1. Clarity of major themes | Yes. These are presented in the three tables, and in the text under the categories of Tourangeau’s model |
| 1. Clarity of minor themes | Yes. These are presented under “other findings”, and reflected on in the discussion. |

1. Tong A, Sainsbury P, Craig J. Consolidated criteria for reporting qualitative research (COREQ): a 32-item checklist for interviews and focus groups. *International journal for quality in health care : journal of the International Society for Quality in Health Care / ISQua* 2007;19(6):349-57. doi: 10.1093/intqhc/mzm042

2. Willis G. Cognitive Interviewing - A How To Guide: Research Triangle Institute, 1999.

3. Tourangeau R. Cognitive sciences and survey methods. In: Jabine T, Straf M, Tanur J, et al., eds. Cognitive Aspects of Survey Methodology: Building a Bridge Between Disciplines. Washington, DC, National Academy Press.1984:73-100.

4. Knäuper B, Carrière K, Chamandy M, et al. How aging affects self-reports. *European Journal of Ageing* 2016;13(2):185-93. doi: 10.1007/s10433-016-0369-0

5. Choi BC, Pak AW. A catalog of biases in questionnaires. *Preventing chronic disease* 2005;2(1):A13.
